# A comparison between neural response telemetry via cochleostomy or the round window approach in cochlear implantation

**DOI:** 10.1590/S1808-86942012000400014

**Published:** 2015-10-20

**Authors:** Rogério Hamerschmidt, Luiz Henrique Schuch, Rodrigo Kopp Rezende, Gislaine Richter Minhoto Wiemes, Adriana Kosma Pires de Oliveira, Marcos Mocellin

**Affiliations:** 1 MSc (Assisting Professor in the Department of Ophthalmology and Otorhinolaryngology at HC/UFPR); 2 MD (Resident Physician, Otorhinolaryngologist); 3 MD, ENT (Otorhinolaryngologist at the Paranaense Institute of Otorhinolaryngology - IPO); 4 MSc (Speech and Hearing Therapist at the Paraná Federal University Hospital - HC/UFPR); 5 MD, ENT (Otorhinolaryngologist at the Paranaense Institute of Otorhinolaryngology); 6 PhD (Professor in the Department of Ophthalmology and Otorhinolaryngology at HC/UFPR); 7 Hospital de Clínicas da Universidade Federal do Paraná

**Keywords:** cochlear implants, round window, ear, telemetry

## Abstract

There are two techniques for cochlear implant (CI) electrode placement: cochleostomy and the round window (RW) approach.

**Objective**: This study aims to compare neural response telemetry (NRT) results immediately after surgery to check for possible differences on auditory nerve stimulation between these two techniques.

**Materials and Methods**: This is a prospective cross-sectional study. Twenty-three patients were enrolled. Six patients underwent surgery by cochleostomy and 17 had it through the RW approach.

**Results**: Mean charge units (MCU) for high frequency sounds: patients submitted to the RW approach had a mean value of 190.4 (±29.2) while cochleostomy patients averaged 187.8 (±32.7); *p* = 0.71. MCU for mid frequency sounds: patients submitted to the RW approach had a mean value of 192.5 (±22) while cochleostomy patients averaged 178.5 (±18.5); *p* = 0.23. MCU for low frequency sounds: patients submitted to the RW approach had a mean value of 183.3 (±25) while cochleostomy patients averaged 163.8 (±19.3); *p* = 0.19.

**Conclusion**: This study showed no differences in the action potential of the distal portion of the auditory nerve in patients with multichannel cochlear implants submitted to surgery by cochleostomy or through the RW approach, using the implant itself to generate stimuli and record responses. Both techniques equally stimulate the cochlear nerve. Therefore, the choice of approach can be made based on the surgeon's own preference and experience.

## INTRODUCTION

Brazil is estimated to have about 347,000 deaf individuals, many of them with indications for a cochlear implant. For patients with little cochlear reserve who can not achieve good sound discrimination even with sound amplification, the cochlear implant (CI) is one option for their rehabilitation^1^. The cochlear implant brings about an improvement in hearing quality and improvements in speech perception and production, rendering a permanent and ascending quality-of-life gain in many aspects - such as self-sufficiency and socialization^2^, ^3^, ^4^, ^5^. It is estimated that since the 70's until today, there are 400 thousand implanted patients^6^.

Cochlear implants partially replace the cochlea by turning sound into electrical signals^7^. The survival of enough neural structures in the cochlear nerve allows the transmission of electric stimuli to the cerebral cortex.

The surgical implantation procedure via the transmastoid approach has been well standardized. Cochleostomy was first described in the 1980s^8^. There are two techniques to place cochlear implants: via a cochleostomy, in which the promontory is drilled to fixate the implant, or via the round window (RW). Less drilling is required in the RW technique, thus reducing trauma, loss of perilymph, and bone powder on the tympanic scale^9^. Preservation of residual hearing has been viable and beneficial due to the combination of electrical and acoustic stimulation, but it requires non-traumatic insertion of the electrode to minimize damage to inner ear structures and enable lesser neural tissue degeneration^6^.

There are different ways to perform objective measurements on the auditory nerves of cochlear implant users from the electrical stimulation of the auditory system such as auditory brainstem response (ABR), middle latency response, late potentials, and stapedial reflexes^1^. Neural response telemetry (NRT) is a test used to measure electrically evoked compound action potentials (ECAP) during surgery or postoperatively in implanted patients. This is an important test used to accurately monitor external and internal hardware function, and assess cochlear stimulation through neural responses^10^.

This is a prospective cross-sectional study aimed at comparing neural response telemetry in the immediate postoperative care of 23 patients of both genders with cochlear implants placed through cochleostomy or the RW approach to verify whether the choice of implantation procedure produces differences in auditory nerve stimulation.

## MATERIALS AND METHODS

This study was approved by the Medical Ethics Committee on Research with Human Subjects and given permit n^o^ 004/2010. This study complied with the standards defined in Resolution 196/96 issued by the Ministry of Health.

Twenty-three patients, seven males and 16 females, were enrolled in this study. Six patients underwent implantation via cochleostomy and 17 via the round window approach. All patients were implanted the same device made by the Cochlear Corporation. The procedures were performed by the same surgeon.

The multichannel cochlear implants used in this study have 22 electrodes placed on the cochlea. The electrodes are numbered from one to twenty-two, 22 being the one placed more apically. These electrodes were grouped the following way: 1-7 high frequency sounds, 8-15 mid-frequency sounds, 16-22 low frequency sounds. This division was needed because during NRT not always we could get neural responses on one same electrode without changing the assessment parameters, and thus we left it for the software program to randomly choose within the groups which electrodes would be analyzed. Electrodes were split by ranges into high, mid, and low frequency groups for the purposes of statistical calculation, as not all electrodes were analyzed individually.

The surgical technique employed to place cochlear implants consists of the following steps: 1. General anesthesia for pediatric patients and local anesthesia plus sedation for adult patients; 2. Retroauricular incision of about three centimeters; 3. Dissection of subcutaneous tissue and muscle plane; 4. Y-shaped periosteal flap; 5. Shift periosteum from the skullcap at the site of implantation of the internal unit; 6. Mastoidectomy; 7. Posterior tympanotomy; 8. Perform cochleostomy in the anterior inferior area of the RW in cases where cochleostomy was used as the implantation approach; drill the upper lip of the round window and open it with a probe; 9. Insert electrode beam; 10. Perform neural response telemetry; and 11. Close the planes of muscle and skin tissue using vicryl 3-0.

All patients were discharged on the same day of surgery and had compressive dressings on for two days. Amoxicillin and clavulanic acid were administered for 10 days. Implants were activated 30 days after surgery.

The Custom Sound AutoNRT measurement system comprises the following elements: 1. A computer with Windows Vista Home Basic, Intel® Pentium® Dual processor; 2. Software version Custom Sound EP 2,0 (2.0.4.7298) and 3,2 (3.2.3855); 3. Programming interface - POD; 4. Speech processor - Freedom sound processor and *headset* SPrint; 5. Freedom Implant (Contour Advance). The NRT software was developed by the Engineering Department at the *Cochlear Corporation*^11^.

A computer equipped with programming interface is used to stimulate specific electrodes inside the cochlea. A series of pulses of information bidirectional communication using encoded radio frequency is transmitted from the Freedom processor interface through an external antenna placed inside a sterile bag placed on the patient's skin above the internal receiver-stimulator. The encoded radio frequency signal controls the stimulation parameters used to evoke compound action potentials. The internal receiver-stimulator in the Freedom Contour cochlear implant is equipped with one amplifier and one analog-digital converter. These additional components allow the voltage recorded on a pair of intracochlear electrodes to be amplified, sampled, and transmitted back to the external antenna, and then to the programming interface. These voltages are analyzed and the resulting ECAP wave is shown on a screen and the data can be stored in a computer. The ECAP records show a negative peak (N1) at 0.2-0.4 ms after stimulus onset, followed by a positive peak (P2) at 0.5-0.7 ms after stimulus onset. Response amplitude is measured from N1 to P2 and ranges between 40-2000 μV. Response amplitude varies with current levels between individuals. The parameters used to measure the thresholds on AutoNRT are: search for thresholds starts at 170 CL, at standard intervals of 6 CL for stimulation levels, at a stimulation frequency of 250 Hz.

## RESULTS

The patients submitted to implantation through the round window approach were aged between 4 and 84 years, and had a mean age of 32 years and three months. Patients in the cochleostomy group were aged between 4 and 54 years, and had a mean age of 19 years.

The Mann-Whitney test was used to statistically treat the samples, as this test allows the comparison of two groups of independent samples of different size.

No statistically significant differences were found between implantation procedures as patients were assessed for high frequency sounds (electrodes 1 to 7) (Table 1).

**Table 1:** Mean current level values for high frequency sounds in patients submitted to implantation via cochleostomy and the round window approach.

Implantation Approach	n	Mean current levels				Mann-Whitney test
		min-max	mean	±	SD	*p*
Round window	17	110-237	190.4	±	29.2	0.71

Cochleostomy	6	146-239	187.8	±	32.7	

n: number of subjects; min-max: minimum and maximum values; SD: standard deviation; *p*: level of statistical significance. Source: designed by Hammerschimt R, Schuch LH and Rezende RK.

No statistically significant differences were found between implantation procedures as patients were assessed for mid-frequency sounds (electrodes 8 to 15) (Table 2).

**Table 2:** Mean current level values for mid-frequency sounds in patients submitted to implantation via cochleostomy and the round window approach.

Implantation Approach	n	Mean current levels				Mann-Whitney test
		min-max	mean	±	SD	*p*
Round window	17	152-236	192.5	±	22.0	0.23

Cochleostomy	6	161-206	178.5	±	18.5	

n: number of subjects; min-max: minimum and maximum values; SD: standard deviation; *p*: level of statistical significance. Source: designed by Hammerschimt R, Schuch LH and Rezende RK.

No statistically significant differences were found between implantation procedures as patients were assessed for low frequency sounds, as seen on Table 3.

**Table 3:** Mean current level values for low frequency sounds in patients submitted to implantation via cochleostomy and the round window approach.

Implantation Approach	n	Mean current levels				Mann-Whitney test
		min-max	mean	±	SD	*p*
Round window	17	134-223	183.3	±	25.0	0.19

Cochleostomy	5	143-190	163.8	±	19.3	

n: number of subjects; min-max: minimum and maximum values; SD: standard deviation; *p*: level of statistical significance. Source: designed by Hammerschimt R, Schuch LH and Rezende RK.

## DISCUSSION

The preservation of the structural tissues of deaf patients is not essential in cochlear implantation procedures. However, since the introduction of electrical and acoustic stimulation combined in patients with cochlear reserve, the preservation of structural tissue and hearing has become of paramount importance in implantation procedures^12^. The loss of residual hearing is the outcome of a combination of factors, including the approach used in the cochleostomy procedure, the electrode neuronal stimuli, and the location of the cochleostomy^8^. The advent of new electrodes and the increased emphasis given to residual hearing preservation have renewed the interest in using the round window as a portal to place electrodes^8^. When compared to cochleostomy via the promontory, placement through the round window should significantly reduce the number of perforation events during electrode placement and thus the risk of trauma, loss of perilymph, and bone powder in the tympanic scale^8^. Bumps around the edges of the round window may pose difficulties to implant placement and require drilling of the anterior inferior border^8^. Drilling in this area must be done carefully, given its proximity to the opening of the cochlear aqueduct^8^.

Each electrode in the cochlear implant has to be programmed so that proper levels of electrical stimulation are provided. The unit used to program electrodes has been arbitrarily chosen and named current level programming units (CL). An important factor concerning cochlear implants is the variation on the current levels needed to elicit hearing for each individual and stimulation channel. Consequently, electrical stimulation parameters must be adjusted on the speech processor for each individual to adapt to specific user needs. This is done through a process called mapping.

A more direct way of measuring cochlear nerve function is electrically evoked compound action potential (ECAP). ECAP reflects the synchronized triggering of cochlear nerve fibers and, in many ways, carried similarities with wave I on ABR, occurring under 0.5 ms after stimulus onset^11^. Originally, these measurements could be done on human beings only during surgery or through cochlear implants using percutaneous stimulation.

Stapedial reflexes can be measured as a response to electrical stimulation of the cochlea through direct observation of stapedial muscle contractions during surgery or by measuring acoustic impedance on the ear contralateral to the implant. The thresholds of electrically evoked stapedial reflex may be used to estimate C levels; nonetheless, significant variability is present in measurements done intra or inter-subjects. Additionally, according to a number of authors these reflexes cannot be recorded in about 40% of the population^13^, ^14^.

Therefore, NRT is a technique that allows ECAP to be measured directly in implanted patients during and after surgery with greater sensitivity, as it can be done in more than 80% of the assessed individuals. NRT is a valuable tool and can be used to confirm the integrity of the internal device, objectively determine which electrodes can be included in a map, define the best stimulation frequencies and speech encoding strategies, estimate T levels to measure the current levels to induce hearing sensation, and estimate C levels - the maximum sensation intensity accepted by patients, a clinically important variable^15^.

No differences were observed on neural response telemetry between implants placed in the tympanic scale via cochleostomy or the round window. One cochleostomy patient had to be excluded from the analysis of mean values for electrodes 16 to 22 as no neural response was captured for low frequency sounds.

Karatas et al.^8^ reported that electrodes placed using the round window approach provided better stimulation when compared to electrodes placed via cochleostomy through the promontory as electrically evoked stapedius reflex thresholds (ESRT) and duration of stimulation were analyzed. In summary, the best response was defined for the shortest response time.

Both cochlear implant placement approaches are well established in the literature, cochleostomy being the most frequently used today. The choice of surgical approach is based on the preferences and training of the surgeon. There are no significant differences between the two approaches in terms of time of surgery and risk of complications.

This paper presents preliminary results, and no analysis was done on neural stimulation to compare patients in different age ranges. The auditory nerves of children respond better to stimulation than the auditory nerves of older patients. In a future study the groups need to be randomized for their specific ages so that this variable is properly assessed for this criterion.

This paper can be used to support other studies with larger samples, specifically on what concerns the cochleostomy approach and the measurement of all electrodes in the immediate postoperative care period. It is also part of the efforts made to reach better results with sound stimulation and auditory rehabilitation of the countless patients affected by deafness.

## CONCLUSION

The data shown in this preliminary study indicates that there is no significant difference in the acquisition of action potentials from the distal portion of the auditory nerve through neural response telemetry in multichannel cochlear implant patients using the implant to elicit stimulation and record responses, whether implantation was done through cochleostomy of the round window approach. Both approaches provide for equal stimulation of the cochlear nerve, and surgeons are free to choose the procedure of their preference.
